# A clinical study on the application of new inflammatory markers SII, SIRI, AISI, and MII in the identification of severe acute pancreatitis

**DOI:** 10.3389/fmed.2026.1880982

**Published:** 2026-09-23

**Authors:** Jingwei Wang, Shujia Chen, Xin Zhao, Jian Lu, Yalong Zhang, Zhengrong Guo

**Affiliations:** 1 Shijiazhuang People's Hospital Affiliated to Hebei Medical University, Shijiazhuang, Hebei, China; 2 Department of Gastroenterology, Shijiazhuang People's Hospital, Shijiazhuang, Hebei, China

**Keywords:** acute pancreatitis, AISI, inflammatory markers, MII, SII, SIRI

## Abstract

**Background:**

Acute pancreatitis (AP) is an inflammatory disease characterized by sudden onset. The mortality rate increases significantly if it progresses to severe acute pancreatitis (SAP). Current clinical scoring systems are complex. Consequently, there is a clinical need for novel inflammatory markers that are inexpensive, fast, accurate, and easy to use. Herein, we aimed to evaluate the clinical value of the following novel inflammatory markers for predicting SAP at admission and assessing AP severity at 48 h after admission: the systemic immune-inflammation index (SII), systemic inflammatory response index (SIRI), aggregate index of systemic inflammation (AISI), multi-inflammatory index-I (MII-1), multi-inflammatory index-II (MII-2), multi-inflammatory index-III (MII-3).

**Methods:**

A retrospective analysis was conducted on 374 patients with AP hospitalized at Shijiazhuang People's Hospital, including 58 cases in the severe group and 316 cases in the non-severe group. Receiver operating characteristic curve analysis and pairwise DeLong tests were used to compare marker performance. Separate multivariable logistic regression models were constructed for each inflammatory marker with adjustment for clinical factors. Internal validation of the adjusted models was performed using 2,000 bootstrap resamples.

**Results:**

The SII, SIRI, AISI, MII-1, MII-2, and MII-3 emerged as statistically significant (*P* < 0.05) indicators of AP severity. At admission, the overall predictive performance of the inflammatory markers for SAP was relatively limited, with AUCs ranging from 0.589 to 0.694; SII showed the highest AUC of 0.694. At 48 h after admission, MII-1, MII-2, and MII-3 showed moderate-to-good discriminatory performance for assessing AP severity, with AUCs of 0.790, 0.778, and 0.780, respectively. After adjustment for clinical factors, all three MII indices remained significantly associated with SAP. The adjusted MII-1, MII-2, and MII-3 models yielded apparent AUCs of 0.819, 0.807, and 0.804 at 48 h after admission, with optimism-corrected AUCs of 0.800, 0.786, and 0.783.

**Conclusion:**

MII-1, MII-2, and MII-3 showed moderate-to-good discriminatory performance for assessing AP severity at 48 h after admission, particularly MII-1, and their associations with SAP remained significant after adjustment for clinical factors. These findings should be considered exploratory and hypothesis-generating, and further studies are needed to validate the potential value of MII indices in AP severity assessment.

## 1 Introduction

Acute pancreatitis (AP) is a common acute gastrointestinal disease, with an estimated global annual incidence of 34 cases per 100,000 people (1). Its pathophysiological characteristics involve localized pancreatic injury triggering a cytokine cascade, which can rapidly progress to systemic inflammation (2). Inflammation plays a role in the onset and progression of AP, which begins as a sterile inflammatory process but may evolve into a bacterial infection in the later stages, accompanied by local and systemic complications. AP has a wide range of nonspecific clinical manifestations, such as mild acute upper abdominal pain, pancreatic and peripancreatic necrosis and infection, systemic inflammatory response syndrome, multi-organ failure, and death (3).

Currently, the most common cause of AP is cholelithiasis, followed by excessive alcohol consumption. Other causes include hypertriglyceridemia, medications, trauma from procedures like endoscopic retrograde cholangiopancreatography (ERCP), hypercalcemia, infection, hereditary diseases, and autoimmune diseases (4–6).

According to the revised Atlanta classification, AP can be categorized into mild acute pancreatitis (MAP), moderately severe acute pancreatitis (MSAP), and severe acute pancreatitis (SAP). MAP is characterized by the absence of local or systemic complications and organ failure. MSAP involves transient organ failure (resolving within 48 h) and/or local or systemic complications. SAP is defined by persistent single- or multi-organ failure (lasting >48 h). The diagnostic criteria for organ failure are based on the modified Marshall scoring system; a score of 2 or higher in any one of the respiratory, cardiovascular, or renal systems indicates a diagnosis of organ failure (7). MAP and MSAP are referred to as non-SAP. Patients with SAP are more likely to develop local or systemic complications and are more likely to progress to chronic pancreatitis (8).

As the clinical manifestations and pathophysiology of AP vary among patients, multiple scoring systems are needed to identify AP severity and predict patient prognosis. Currently used tools include the Ranson score, Acute Physiology and Chronic Health Evaluation II (APACHE II), Bedside Index for Severity in Acute Pancreatitis (BISAP), Sequential Organ Failure Assessment (SOFA), Modified Computed Tomography Severity Index (MCTSI), and the Balthazar grade (1, 9, 10). The Ranson score has low sensitivity in the early stages and a complex data collection process, involving 11 indicators, hindering its application. The APACHE II score has long been validated for predicting in-hospital mortality risk in critically ill patients; however, this index encompasses 12 indicators, which makes data collection and statistical calculations complex, limiting its suitability to intensive care unit patients and rendering it inapplicable to most patients. BISAP is a scoring system used for simple assessment of disease severity and mortality risk; however, it cannot distinguish between edematous and necrotizing pancreatitis. SOFA is primarily used for patients with sepsis to evaluate the progression of multi-organ dysfunction in critically ill patients. The MCTSI and the Balthazar grade are derived from radiological imaging and used to assess AP severity. However, as they do not incorporate laboratory parameters, they cannot reflect the patient's overall condition or the specific extent and number of organ failures (1, 10, 11). Taken together, although the aforementioned scoring systems have proven clinical value, their limitations—including extensive data requirements pertaining to numerous laboratory parameters and the complexity of their application—make them costly and unsuitable for primary care hospitals, hindering their widespread implementation. Therefore, identifying inexpensive, fast, accurate, and easy to use clinical indicators is crucial for the assessment of disease severity and risk stratification in AP. Several laboratory parameter-based indices—specifically systemic immune-inflammation index (SII), systemic inflammatory response index (SIRI), aggregate index of systemic inflammation (AISI), multi-inflammatory index-Ⅰ (MII-1), multi-inflammatory index-Ⅱ (MII-2), and multi-inflammatory index-Ⅲ (MII-3)—are used to assess the severity of inflammation and prognosis of various diseases, such as cancer (12–14), myocardial infarction (15), atrial fibrillation following coronary artery bypass grafting (16, 17), ischemic stroke (18, 19), pulmonary embolism (20), severe COVID-19 (21), and Crimean-Congo hemorrhagic fever (CCHF) (22), among others.

Conventional inflammatory indices, such as the neutrophil-to-lymphocyte ratio (NLR) and platelet-to-lymphocyte ratio (PLR), have also been investigated for the assessment of AP severity (2, 9). In the present study, C-reactive protein (CRP), NLR, and PLR were additionally evaluated as reference inflammatory markers for a more comprehensive comparative assessment. This comparison was particularly relevant for MII indices, as MII-1, MII-2, and MII-3 are calculated from NLR × CRP, PLR × CRP, and SII × CRP, respectively. The severity of AP is not fully quantifiable at admission; as the condition evolves, its severity becomes apparent. The 48-h mark after onset is a critical time point to identify whether the local inflammatory response is transitioning to a systemic one; it also serves as a key juncture for adjusting treatment strategies and assessing prognosis (1, 3).

This study aims to evaluate the clinical value of novel inflammatory markers (SII, SIRI, AISI, MII-1, MII-2, and MII-3) for predicting SAP at admission and assessing AP severity at 48 h after admission, with CRP, NLR, and PLR included as reference inflammatory markers for comparative evaluation.

## 2 Materials and methods

### 2.1 Study design

This single-center retrospective study initially screened 461 patients with AP who were hospitalized at Shijiazhuang People's Hospital between April 1, 2023 and February 1, 2026. This study was approved by the Ethics Committee of Shijiazhuang People's Hospital (approval no.: 2026050).

The inclusion criteria were as follows: age ≥ 18 years and meeting at least two of the following three diagnostic criteria for AP: (i) acute persistent upper abdominal pain, (ii) serum amylase or lipase levels at least three times the upper limit of normal, and (iii) imaging findings consistent with AP on ultrasonography (US), computed tomography, or magnetic resonance imaging. The exclusion criteria were as follows: age <18 years; severe dysfunction of the heart, lungs, kidneys, liver, or other organs (including end-stage liver or kidney disease); hypersplenism; pregnancy; hospital stay shorter than 48 h; missing key clinical data; and history of malignancy, partial pancreatectomy, or hereditary or acquired bone marrow involvement (e.g., aplastic anemia, myelodysplastic syndrome, primary immune thrombocytopenia, multiple myeloma, acute myeloid leukemia, chronic myeloid leukemia, acute lymphocytic leukemia, chronic lymphocytic leukemia, and lymphoma, among others).

Among the 461 patients initially screened, 87 were excluded according to the predefined exclusion criteria: 41 with missing key clinical data, 16 with a history of malignancy, 8 with hypersplenism, 6 aged <18 years, 6 with a hospital stay shorter than 48 h, 5 with severe cardiopulmonary dysfunction, 2 with severe renal dysfunction due to end-stage renal disease, 2 with a history of partial pancreatectomy, and 1 with hereditary or acquired bone marrow involvement. Finally, 374 patients were included in the analysis, including 58 patients with SAP and 316 patients with non-SAP.

Following review of the complete hospitalization records, the study investigators retrospectively assessed and classified AP severity according to the revised Atlanta classification. Organ failure was defined as a score of 2 or higher in any of the respiratory, cardiovascular, or renal systems of the modified Marshall scoring system. Patients without organ failure or local or systemic complications were classified as having MAP. MSAP was classified by transient organ failure resolving within 48 h or local or systemic complications in the absence of persistent organ failure or both, whereas persistent single- or multiorgan failure lasting >48 h was classified as SAP (7). For the present analysis, MAP and MSAP were combined into the non-SAP group (8). Severity classification was completed before the derived inflammatory indices (SII, SIRI, AISI, MII-1, MII-2, and MII-3) were calculated; therefore, these index values were not available to the investigators at the time of severity classification.

### 2.2 Data collection

Comprehensive clinical data were collected at the time of admission and 48 h after admission. Demographic information was collected, including sex, age, body mass index (BMI), smoking, alcohol consumption, etiology of AP (biliary, hyperlipidemic, alcoholic, and others), and length of hospital stay (LOS). The following laboratory parameters were collected: white blood cell count (WBC), neutrophils, platelets, lymphocytes, monocytes, CRP, hematocrit (HCT), blood glucose (Glu), albumin (ALB), total cholesterol (TC), triglycerides (TG), creatinine (CREA), blood urea nitrogen (BUN), potassium (K+), sodium (Na+), alanine aminotransferase (ALT), aspartate aminotransferase (AST), alkaline phosphatase (ALP), gamma-glutamyl transferase (GGT), total bilirubin (TBIL), and direct bilirubin (DBIL).

### 2.3 Calculation of inflammatory markers

The primary inflammatory markers evaluated in this study were SII, SIRI, AISI, MII-1, MII-2, and MII-3. CRP, NLR, and PLR were additionally included as reference inflammatory markers in the subsequent comparative analyses. The inflammatory markers were calculated as follows:

NLR: Neutrophil count/Lymphocyte count,
PLR: Platelet count/Lymphocyte count,
SII: Neutrophil count × Platelet count/Lymphocyte count,
SIRI: Neutrophil count × Monocyte count/Lymphocyte count,
AISI: Neutrophil count × Monocyte count × Platelet count/Lymphocyte count,
MII-1: Neutrophil count/Lymphocyte count × C-reactive protein,
MII-2: Platelet count/Lymphocyte count × C-reactive protein, and
MII-3: SII × C-reactive protein.

### 2.4 Statistical analysis

Data analysis was performed using the IBM SPSS Statistics version 31 program (International Business Machines Corporation, Armonk, New York, USA) and R version 4.6.1 (R Foundation for Statistical Computing, Vienna, Austria). Missing data were uncommon in the final study population. BMI was missing in 7 of 374 patients (1.87%), CRP at admission in 3 patients (0.80%), and both TC and TG were missing in the same 4 patients (1.07%). Missing values were handled using multiple imputation under the assumption that the data were missing at random (MAR). The number of imputations was set to 10. The imputation model included all variables used in the subsequent analyses, including SAP status and all predictor variables. As SAP status was complete for all patients, it was included only as a predictor in the imputation model. Continuous variables following a normal distribution were expressed as mean ± standard deviation, and between-group comparisons were performed using the t-test; non-normally distributed continuous variables were expressed as median and interquartile range, and between-group comparisons were performed using the Mann–Whitney U test. Categorical variables were expressed as frequencies and percentages, and between-group comparisons were performed using the Chi-square test.

Spearman correlation analyses were performed separately at admission and 48 h after admission to assess pairwise correlations among CRP, NLR, PLR, SII, SIRI, AISI, MII-1, MII-2, and MII-3. Receiver operating characteristic (ROC) curve analyses were performed separately for CRP, NLR, PLR, SII, SIRI, AISI, MII-1, MII-2, and MII-3 to evaluate the predictive performance of the inflammatory markers for SAP at admission and their discriminatory performance for assessing AP severity at 48 h after admission. For each marker, the area under the curve (AUC), sensitivity, specificity, and optimal cutoff value were calculated. The 95% CIs for the AUCs were estimated using 2,000 stratified bootstrap resamples, and optimal cutoff values were determined by maximizing the Youden index (sensitivity + specificity−1). Pairwise comparisons of correlated ROC curves were performed using the DeLong test. Each MII index (MII-1, MII-2, and MII-3) was compared with CRP, NLR, PLR, SII, SIRI, and AISI at each time point. *Δ*AUC was calculated as the AUC of the MII index minus that of the comparator (23).

Given the limited number of patients with SAP (*n* = 58), model complexity was intentionally restricted to minimize the risk of overfitting. Multivariable logistic regression models were constructed with SAP status as the dependent variable. Separate models were constructed for each inflammatory marker at each time point, with adjustment for age, sex, BMI, alcohol consumption, and triglyceride levels. Triglyceride levels and inflammatory markers were log₂-transformed before modeling. Sex was coded as male = 1 and female = 0, and alcohol consumption was coded as yes = 1 and no = 0. Adjusted odds ratios (ORs) and 95% confidence intervals (CIs) were evaluated. For the inflammatory markers, ORs represent the change in the odds of SAP per doubling of the corresponding marker. Multicollinearity in the multivariable logistic regression models was assessed using variance inflation factors (VIFs), and the maximum VIF for each model was reported.

ROC curves were also generated for the adjusted multivariable logistic regression models to evaluate their predictive performance for SAP at admission and their discriminatory performance for assessing AP severity at 48 h after admission. Internal validation of these models was performed using 2,000 nonparametric bootstrap resamples. In each bootstrap resample, the model was refitted using the same variables as in the original model. Optimism-corrected AUCs and calibration slopes were obtained by subtracting the mean bootstrap-estimated optimism for each performance measure from the corresponding apparent estimates. ROC analyses for individual inflammatory markers and multivariable models were performed using the *pROC* package in R. Bootstrap confidence intervals for individual marker AUCs and pairwise DeLong tests were likewise implemented using *pROC* (24). A *P* value of <0.05 was considered statistically significant.

## 3 Results

This study included 374 patients [260 males (69.5%) and 114 females (30.5%)] meeting the inclusion criteria between April 1, 2023 and February 1, 2026; among them, 58 were in the severe group and 316 were in the non-severe group. Their demographic and clinical characteristics are shown in Table 1. The two groups were comparable in terms of age, with a mean age of 43 years (range, 35–62 years, *P* = 0.840), and sex distribution (*P* = 0.921). The severe group had a significantly higher BMI than the non-severe group (*P* = 0.002), indicating that a higher BMI may increase the risk of developing SAP. The LOS was significantly longer in the severe group [median, 11.50 days (range, 9.75–16.25 days)] than in the non-severe group [median, 9 days (range, 7–11 days); *P* < 0.001]. The proportion of patients with a history of alcohol consumption, a primary cause of AP, was significantly higher in the severe group (51.7%) than in the non-severe group (36.7%; *P* = 0.031); however, the etiology did not significantly differ between the two groups (*P* = 0.479). The incidence of smoking did not statistically significantly differ between the two groups (*P* = 0.080). TG levels were significantly higher in the severe group than in the non-severe group (*P* = 0.025). The following did not statistically significantly differ between the groups: ALB, ALT, AST, GGT, ALP, TBIL, DBIL, BUN, HCT, CREA, TC, Na+, K+, or Glu.

**Table 1:** Comparison of demographic and clinical characteristics between patient groups.

Variables	Non-severe group (*n* = 316)	Severe group (*n* = 58)	*P* value
Age [years, median (IQR)]	43.00 (35.00–62.00)	40.50 (35.00–62.75)	0.840
Sex (*n*, %)			0.921
Male	220 (69.6)	40 (69.0)	
Female	96 (30.4)	18 (31.0)	
BMI [kg/m^2^_,_ median (IQR)]	26.25 (23.44–29.39)	28.69 (25.34–31.89)	0.002
LOS [days, median (IQR)]	9.00 (7.00–11.00)	11.50 (9.75–16.25)	<0.001
Etiology (*n*, %)			0.479
Biliary	147 (46.5)	24 (41.4)	
Hyperlipidemic	74 (23.4)	18 (31.0)	
Alcoholic	35 (11.1)	8 (13.8)	
Others	60 (19.0)	8 (13.8)	
Smoking (*n*, %)			0.080
No	212 (67.1)	32 (55.2)	
Yes	104 (32.9)	26 (44.8)	
Alcohol consumption (*n*, %)			0.031
No	200 (63.3)	28 (48.3)	
Yes	116 (36.7)	30 (51.7)	
ALB [g/L, median (IQR)]	40.85 (36.40–44.40)	41.25 (35.75–44.95)	0.888
ALT [U/L, median (IQR)]	40.00 (23.00–115.50)	43.00 (22.50–141.50)	0.492
AST [U/L, median (IQR)]	27.00 (17.00–83.50)	34.00 (19.00–143.25)	0.125
GGT [U/L, median (IQR)]	86.00 (37.25–258.00)	106.00 (39.75–349.00)	0.338
ALP [U/L, median (IQR)]	86.00 (69.00, 121.00)	90.00 (70.50–120.25)	0.658
TBIL [µmol/L, median (IQR)]	20.45 (13.90–35.67)	23.35 (14.47–40.20)	0.271
DBIL [µmol/L, median (IQR)]	4.60 (2.23–12.28)	5.60 (2.68–16.30)	0.353
BUN [mmol/L, median (IQR)]	2.10 (1.70–2.88)	2.30 (1.98–3.08)	0.054
HCT (%, mean ± SD)	42.78 ± 5.07	43.44 ± 6.29	0.452
CREA [µmol/L, median (IQR)]	63.00 (51.00–77.00)	62.00 (45.00–93.00)	0.859
TC [mmol/L, median (IQR)]	4.98 (3.86–7.13)	5.63 (3.84–8.83)	0.186
TG [mmol/L, median (IQR)]	2.40 (0.93–10.69)	4.08 (1.38–24.18)	0.025
Na^+^ [mmol/L, median (IQR)]	136.20 (133.20–138.50)	135.80 (132.07–138.72)	0.567
K^+^ [mmol/L, median (IQR)]	3.87 (3.62–4.14)	3.83 (3.48–4.14)	0.336
Glu [mmol/L, median (IQR)]	8.50 (6.60–11.80)	9.65 (7.20–13.38)	0.074

BMI, body mass index; LOS, length of hospital stay; ALB, albumin; ALT, alanine aminotransferase; AST, aspartate aminotransferase; GGT, gamma-glutamyl transferase; ALP, alkaline phosphatase; TBIL, total bilirubin; DBIL, direct bilirubin; BUN, blood urea nitrogen; HCT, hematocrit; CREA, creatinine; TC, total cholesterol; TG, triglycerides; Na^+^, sodium; K^+^, potassium; Glu, blood glucose.

Between-group comparisons at admission and 48 h after admission showed that compared to the non-severe group, the severe group had significantly higher neutrophil counts, CRP, NLR, PLR levels and the SII, SIRI, AISI, MII-1, MII-2, and MII-3 values but significantly lower lymphocyte counts (*P* < 0.05). Platelet and monocyte counts did not differ significantly between the two groups (Tables 2, 3).

**Table 2:** Comparison of inflammatory markers between patient groups at admission.

Variables	Non-severe group (*n* = 316)	Severe group (*n* = 58)	*P* value
Neut [ × 10^9^/L, median (IQR)]	9.51 (7.27–12.65)	11.21 (10.09–14.23)	<0.001
Ly [ × 10^9^/L, median (IQR)]	1.16 (0.76–1.70)	0.96 (0.65–1.25)	0.008
Mon [ × 10^9^/L, median (IQR)]	0.56 (0.40–0.76)	0.64 (0.37–0.86)	0.280
PLT [ × 10^9^/L, median (IQR)]	229.50 (180.25–267.00)	244.00 (186.00–306.50)	0.147
CRP [mg/L, median (IQR)]	16.21 (3.75–87.40)	31.50 (7.22–151.68)	0.030
NLR [median (IQR)]	8.46 (5.12–13.46)	12.91 (9.19–19.20)	<0.001
PLR [median (IQR)]	191.78 (129.55–294.94)	267.53 (177.48–405.23)	0.001
SII [median (IQR)]	1,853.52 (1,046.46–3,169.09)	3,409.74 (1,929.22–5,237.99)	<0.001
SIRI [median (IQR)]	4.58 (2.56–7.95)	6.85 (4.08–12.18)	<0.001
AISI [median (IQR)]	1,012.53 (514.18–1,884.47)	1,696.75 (874.09–2,711.81)	<0.001
MII-1 [median (IQR)]	138.29 (22.14–912.62)	496.29 (118.91–1,939.58)	0.001
MII-2 [median (IQR)]	3,300.28 (678.12–19,609.49)	7,815.64 (2,265.51–39,106.76)	0.004
MII-3 [median (IQR)]	31,168.40 (5,330.59–1,99,318.89)	86,637.41 (32,071.51–4,39,771.06)	<0.001

Neut, neutrophil count; Ly, lymphocyte count; Mon, monocyte count; PLT, platelet count; CRP, C-reactive protein; NLR, neutrophil to lymphocyte ratio; PLR, platelet to lymphocyte ratio; SII, systemic immune-inflammation index; SIRI, systemic inflammatory response index; AISI, aggregate index of systemic inflammation; MII-1, multi-inflammatory index-Ⅰ; MII-2, multi-inflammatory index-II; MII-3, multi-inflammatory index-III.

**Table 3:** Comparison of inflammatory markers between groups at 48 h after admission.

Variables	Non-severe group (*n* = 316)	Severe group (*n* = 58)	*P* value
Neut [ × 10^9^/L, median (IQR)]	7.12 (4.68–9.34)	10.11 (7.25–13.04)	<0.001
Ly [×10^9^/L, median (IQR)]	1.21 (0.93–1.63)	0.95 (0.73–1.23)	<0.001
Mon [×10^9^/L, median (IQR)]	0.56 (0.42–0.78)	0.66 (0.41–0.79)	0.349
PLT [×10^9^/L, median (IQR)]	198.00 (156.25–239.75)	194.00 (159.50–243.50)	0.976
CRP [mg/L, median (IQR)]	115.34 (49.87–188.55)	229.86 (149.76–291.50)	<0.001
NLR [median (IQR)]	5.55 (3.28–9.20)	10.37 (7.18–13.64)	<0.001
PLR [median (IQR)]	155.69 (112.61–214.85)	217.31 (141.80–277.10)	<0.001
SII [median (IQR)]	1,009.35 (570.50–1,784.67)	2,047.22 (1,388.78–2,954.20)	<0.001
SIRI [median (IQR)]	3.38 (1.60–6.25)	6.04 (3.52–12.07)	<0.001
AISI [median (IQR)]	576.33 (275.54–1,239.97)	1,335.74 (648.19–2,174.44)	<0.001
MII-1 [median (IQR)]	679.36 (210.45–1,573.30)	2,584.64 (1,243.36–3,457.14)	<0.001
MII-2 [median (IQR)]	18,268.67 (7,014.24–32,355.58)	41,439.96 (25,842.32–68,261.44)	<0.001
MII-3 [median (IQR)]	1,23,193.73 (32,261.95–2,89,821.38)	4,73,558.11 (2,04,200.62–8,29,854.06)	<0.001

Neut, neutrophil count; Ly, lymphocyte count; Mon, monocyte count; PLT, platelet count; CRP, C-reactive protein; NLR, neutrophil to lymphocyte ratio; PLR, platelet to lymphocyte ratio; SII, systemic immune-inflammation index; SIRI, systemic inflammatory response index; AISI, aggregate index of systemic inflammation; MII-1, multi-inflammatory index-Ⅰ; MII-2, multi-inflammatory index-II; MII-3, multi-inflammatory index-III.

Spearman correlation analysis revealed significant positive correlations among most inflammatory markers at admission and 48 h after admission (Supplementary Tables S1 and S2). At admission, all pairwise correlations were statistically significant, except for that between CRP and PLR (rs = 0.099). Strong positive correlations were observed among MII-1, MII-2, and MII-3, with correlation coefficients ranging from 0.981 to 0.988, as well as between CRP and the three MII markers, with correlation coefficients ranging from 0.931 to 0.953. At 48 h after admission, all pairwise correlations were statistically significant. Strong positive correlations persisted among MII-1, MII-2, and MII-3, with coefficients ranging from 0.912 to 0.963, while the correlations between CRP and MII markers weakened, with coefficients ranging from 0.866 to 0.872.

ROC curves were plotted to evaluate the predictive performance of the individual inflammatory markers for SAP at admission and their discriminatory performance for assessing AP severity at 48 h after admission, as shown in Tables 4, 5 and Figures 1, 2. At admission, the overall predictive performance of the inflammatory markers was relatively limited, with AUCs ranging from 0.589 to 0.694. SII showed the highest AUC (AUC = 0.694, 95% CI: 0.620–0.761, *P* < 0.001), followed by NLR (AUC = 0.686, 95% CI: 0.618–0.753, *P* < 0.001). The AUCs of MII-1, MII-2, and MII-3 were 0.641 (95% CI: 0.572–0.708, *P* < 0.001), 0.620 (95% CI: 0.547–0.694, *P* = 0.002), and 0.648 (95% CI: 0.578–0.716, *P* < 0.001), respectively. At 48 h after admission, the three MII markers showed moderate-to-good discriminatory performance for assessing AP severity and yielded the three highest AUCs among the evaluated inflammatory markers. MII-1 showed the highest AUC (AUC = 0.790, 95% CI: 0.728–0.845, *P* < 0.001), followed by MII-3 (AUC = 0.780, 95% CI: 0.717–0.840, *P* < 0.001) and MII-2 (AUC = 0.778, 95% CI: 0.718–0.835, *P* < 0.001). At the optimal cutoff value of 1,519.06, MII-1 had a sensitivity of 0.741 and specificity of 0.744.

**Table 4:** ROC curve analysis of individual inflammatory markers at admission.

Variables	AUC	95% CI	Cut-off	Sensitivity	Specificity	Youden index	*P* value
CRP	0.589	0.511–0.667	2.22	0.966	0.190	0.155	0.024
NLR	0.686	0.618–0.753	8.37	0.845	0.491	0.335	<0.001
PLR	0.632	0.554–0.707	175.92	0.776	0.465	0.241	<0.001
SII	0.694	0.620–0.761	3,285.95	0.534	0.785	0.319	<0.001
SIRI	0.657	0.585–0.731	3.71	0.828	0.418	0.245	<0.001
AISI	0.653	0.577–0.727	744.34	0.862	0.396	0.258	<0.001
MII-1	0.641	0.572–0.708	94.67	0.810	0.453	0.263	<0.001
MII-2	0.620	0.547–0.694	2,444.59	0.759	0.468	0.227	0.002
MII-3	0.648	0.578–0.716	26,676.29	0.793	0.487	0.280	<0.001

ROC, receiver operating characteristic; AUC, area under the curve; CI, confidence interval; CRP, C-reactive protein; NLR, neutrophil to lymphocyte ratio; PLR, platelet to lymphocyte ratio; SII, systemic immune-inflammation index; SIRI, systemic inflammatory response index; AISI, aggregate index of systemic inflammation; MII-1, multi-inflammatory index-Ⅰ; MII-2, multi-inflammatory index-II; MII-3, multi-inflammatory index-III.

The 95% CIs for the AUCs were estimated using 2,000 stratified bootstrap resamples. Optimal cutoff values were determined by maximizing the Youden index, defined as sensitivity + specificity−1.

**Table 5:** ROC curve analysis of individual inflammatory markers at 48 h after admission.

Variables	AUC	95% CI	Cut-off	Sensitivity	Specificity	Youden index	*P* value
CRP	0.758	0.695–0.816	187.86	0.672	0.750	0.422	<0.001
NLR	0.742	0.680–0.798	5.52	0.914	0.500	0.414	<0.001
PLR	0.645	0.567–0.722	211.75	0.534	0.747	0.281	<0.001
SII	0.734	0.662–0.799	1,661.18	0.724	0.725	0.449	<0.001
SIRI	0.700	0.628–0.767	2.43	0.931	0.421	0.352	<0.001
AISI	0.698	0.625–0.767	858.71	0.707	0.633	0.340	<0.001
MII-1	0.790	0.728–0.845	1,519.06	0.741	0.744	0.485	<0.001
MII-2	0.778	0.718–0.835	20,589.17	0.879	0.563	0.443	<0.001
MII-3	0.780	0.717–0.840	2,42,804.44	0.741	0.712	0.453	<0.001

ROC, receiver operating characteristic; AUC, area under the curve; CI, confidence interval; CRP, C-reactive protein; NLR, neutrophil to lymphocyte ratio; PLR, platelet to lymphocyte ratio; SII, systemic immune-inflammation index; SIRI, systemic inflammatory response index; AISI, aggregate index of systemic inflammation; MII-1, multi-inflammatory index-Ⅰ; MII-2, multi-inflammatory index-II; MII-3, multi-inflammatory index-III.

The 95% CIs for the AUCs were estimated using 2,000 stratified bootstrap resamples. Optimal cutoff values were determined by maximizing the Youden index, defined as sensitivity + specificity−1.

**Figure 1:**
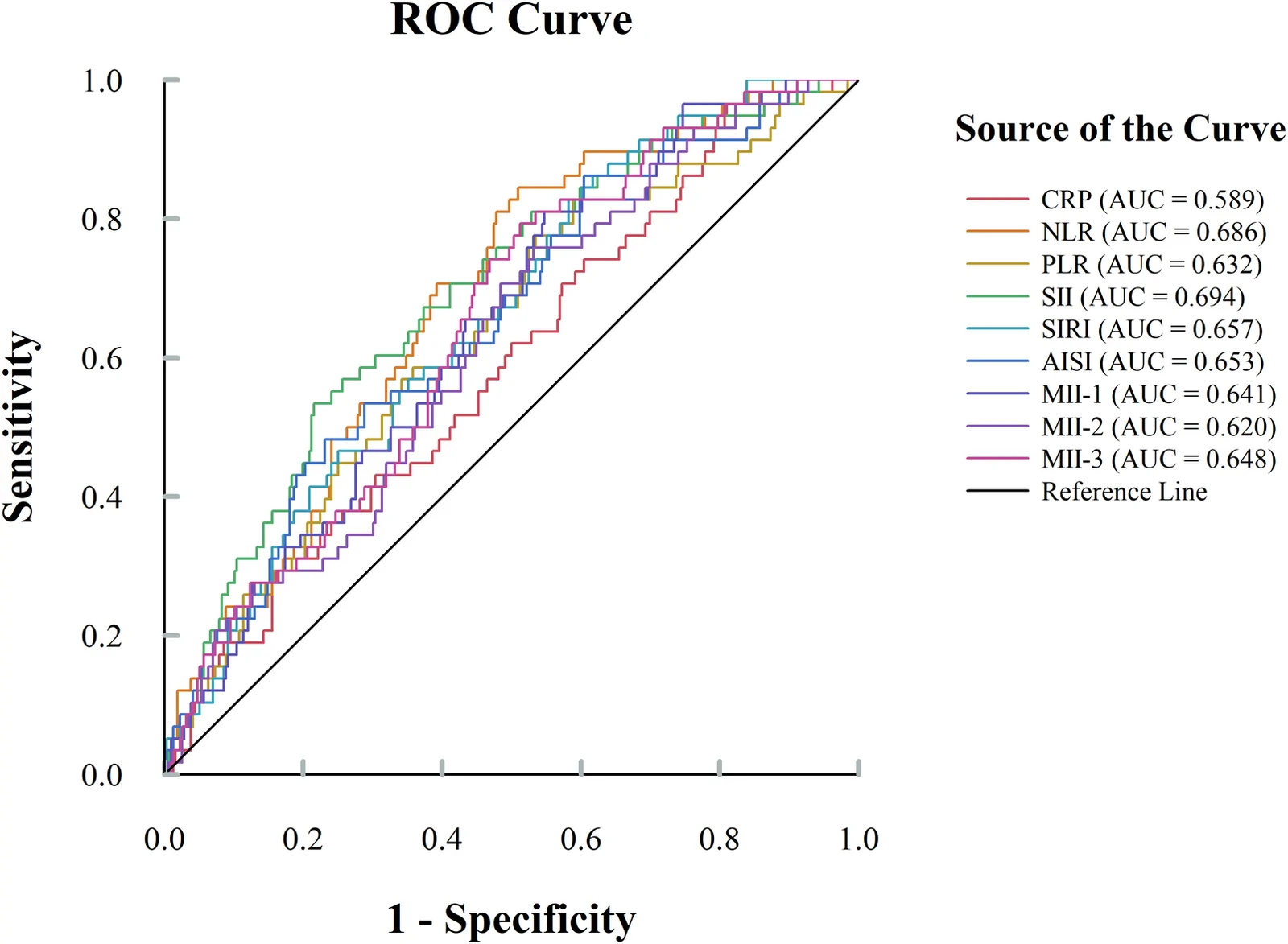
ROC curves for various inflammatory markers for predicting SAP at admission. ROC, receiver operating characteristic; CRP, C-reactive protein; NLR, neutrophil to lymphocyte ratio; PLR, platelet to lymphocyte ratio; SII, systemic immune-inflammation index; SIRI, systemic inflammatory response index; AISI, aggregate index of systemic inflammation; MII-1, multi-inflammatory index-I; MII-2, multi-inflammatory index-II; MII-3, multi-inflammatory index-III.

**Figure 2:**
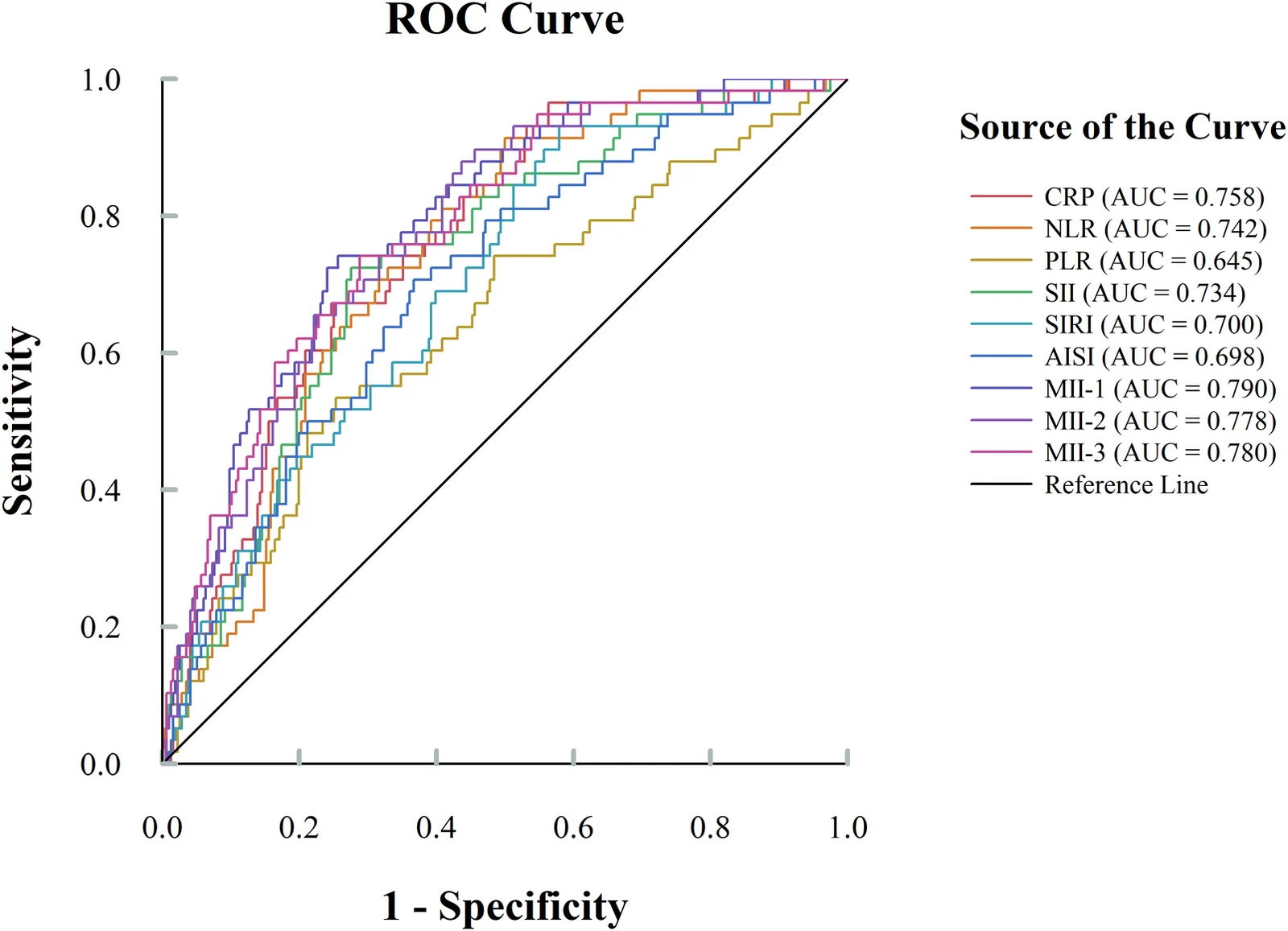
ROC curves for various inflammatory markers for assessing AP severity at 48 h after admission. ROC, receiver operating characteristic; CRP, C-reactive protein; NLR, neutrophil to lymphocyte ratio; PLR, platelet to lymphocyte ratio; SII, systemic immune-inflammation index; SIRI, systemic inflammatory response index; AISI, aggregate index of systemic inflammation; MII-1, multi-inflammatory index-I; MII-2, multi-inflammatory index-II; MII-3, multi-inflammatory index-III.

Pairwise DeLong comparisons were further performed to compare the performance of MII markers with that of other inflammatory markers at each time point. At admission, MII-1, MII-2, and MII-3 showed significantly higher AUCs than CRP (*Δ*AUC = 0.052, *P* < 0.001; *Δ*AUC = 0.031, *P* = 0.024; and *Δ*AUC = 0.058, *P* < 0.001, respectively), whereas no significant differences were observed between MII markers and NLR, PLR, SII, SIRI, or AISI (Supplementary Table S3).

At 48 h after admission, MII-1 showed significantly higher AUCs than NLR, PLR, SII, SIRI, and AISI; MII-2 showed significantly higher AUCs than PLR, SIRI, and AISI; and MII-3 showed significantly higher AUCs than PLR, SII, SIRI, and AISI (all *P* < 0.05; Supplementary Table S4). No significant differences were observed between any of the three MII markers and CRP (all *P* > 0.05). Notably, compared with their corresponding inflammatory indices, MII-1 showed a significantly higher AUC than NLR (*Δ*AUC = 0.048, *P* = 0.017), MII-2 than PLR (*Δ*AUC = 0.132, *P* < 0.001), and MII-3 than SII (*Δ*AUC = 0.047, *P* = 0.002), indicating improved discriminatory performance of MII markers over their corresponding indices for assessing AP severity at 48 h after admission (Table 6).

**Table 6:** Pairwise DeLong comparisons of MII-1, MII-2, and MII-3 With CRP and their corresponding inflammatory markers at 48 h after admission.

Variables marker	AUC of MII	Comparator	AUC of comparator	*Δ*AUC	*P* value
MII-1	0.790	CRP	0.758	0.032	0.119
MII-1	0.790	NLR	0.742	0.048	0.017
MII-2	0.778	CRP	0.758	0.020	0.370
MII-2	0.778	PLR	0.645	0.132	<0.001
MII-3	0.780	CRP	0.758	0.022	0.245
MII-3	0.780	SII	0.734	0.047	0.002

AUC, area under the curve; CRP, C-reactive protein; NLR, neutrophil to lymphocyte ratio; PLR, platelet to lymphocyte ratio; SII, systemic immune-inflammation index; SIRI, systemic inflammatory response index; AISI, aggregate index of systemic inflammation; MII-1, multi-inflammatory index-Ⅰ; MII-2, multi-inflammatory index-II; MII-3, multi-inflammatory index-III.

*Δ*AUC was calculated as the AUC of the MII index minus that of the comparator. *P* values were obtained using paired DeLong tests for correlated ROC curves. A positive *Δ*AUC indicates better discrimination by the MII marker than by the comparator.

After adjustment for age, sex, BMI, alcohol consumption, and triglyceride levels, multivariable logistic regression analysis showed that—at admission—CRP was not significantly associated with SAP (OR: 1.083, 95% CI: 0.996–1.214, *P* = 0.173), whereas NLR, PLR, SII, SIRI, and AISI were significantly associated with SAP (all *P* < 0.001). MII-1 (OR: 1.157, 95% CI: 1.043–1.283, *P* = 0.006), MII-2 (OR: 1.132, 95% CI: 1.018–1.259, *P* = 0.022), and MII-3 (OR: 1.165, 95% CI: 1.051–1.292, *P* = 0.004) were also significantly associated with SAP. At 48 h after admission, all inflammatory markers were significantly associated with SAP (all *P* < 0.001). For MII markers, the ORs were 2.067 (95% CI: 1.613–2.649, *P* < 0.001) for MII-1, 2.203 (95% CI: 1.648–2.945, *P* < 0.001) for MII-2, and 1.747 (95% CI: 1.431–2.134, *P* < 0.001) for MII-3 (Table 7). The regression coefficients for all variables included in the separate multivariable logistic regression models at admission and 48 h after admission are provided in Supplementary Tables S5 and S6.

**Table 7:** Multivariable logistic regression analysis of the association between inflammatory markers and AP severity after adjustment for clinical factors.

Variables	At admission		At 48 h After admission	
	*P* value	Adjusted OR (95% CI)	*P* value	Adjusted OR (95% CI)
CRP	0.173	1.083 (0.996–1.214)	<0.001	2.543 (1.692–3.822)
NLR	<0.001	2.125 (1.539–2.936)	<0.001	3.268 (2.226–4.799)
PLR	<0.001	1.854 (1.312–2.619)	<0.001	2.332 (1.512–3.597)
SII	<0.001	1.929 (1.449–2.569)	<0.001	2.129 (1.601–2.830)
SIRI	<0.001	1.561 (1.229–1.982)	<0.001	1.972 (1.512–2.572)
AISI	<0.001	1.463 (1.188–1.802)	<0.001	1.645 (1.324–2.044)
MII-1	0.006	1.157 (1.043–1.283)	<0.001	2.067 (1.613–2.649)
MII-2	0.022	1.132 (1.018–1.259)	<0.001	2.203 (1.648–2.945)
MII-3	0.004	1.165 (1.051–1.292)	<0.001	1.747 (1.431–2.134)

OR, odds ratio; CI, confidence interval; CRP, C-reactive protein; NLR, neutrophil to lymphocyte ratio; PLR, platelet to lymphocyte ratio; SII, systemic immune-inflammation index; SIRI, systemic inflammatory response index; AISI, aggregate index of systemic inflammation; MII-1, multi-inflammatory index-Ⅰ; MII-2, multi-inflammatory index-II; MII-3, multi-inflammatory index-III.

All models were adjusted for age, sex, BMI, alcohol consumption, and log_2_-transformed triglyceride levels, with each log_2_-transformed inflammatory marker evaluated in a separate model. The ORs for inflammatory markers represent the change in the odds of SAP per doubling of the corresponding marker.

No substantial multicollinearity was observed in the multivariable logistic regression models, with maximum VIFs ranging from 1.420 to 1.540 at admission and from 1.424 to 1.555 at 48 h after admission (Supplementary Table S7).

ROC analysis of the adjusted multivariable models showed relatively modest predictive performance for SAP at admission, with apparent AUCs ranging from 0.697 to 0.770. The NLR model showed the highest apparent AUC of 0.770, followed by the SII model, showing an AUC of 0.767. After bootstrap internal validation, the optimism-corrected AUCs ranged from 0.659 to 0.744, whereas the optimism-corrected calibration slopes ranged from 0.783 to 0.856. The optimism-corrected AUCs for the MII-1, MII-2, and MII-3 models were 0.692, 0.680, and 0.697, respectively (Table 8 and Figure 3).

**Table 8:** Bootstrap internal validation of the adjusted models at admission.

Variables	Apparent AUC	Estimated optimism	Optimism-corrected AUC	Optimism-corrected calibration slope
CRP	0.697	0.038	0.659	0.783
NLR	0.770	0.026	0.744	0.856
PLR	0.736	0.031	0.705	0.827
SII	0.767	0.025	0.742	0.853
SIRI	0.729	0.030	0.699	0.843
AISI	0.732	0.031	0.701	0.833
MII-1	0.723	0.031	0.692	0.821
MII-2	0.713	0.034	0.680	0.810
MII-3	0.728	0.030	0.697	0.826

AUC, area under the curve; CRP, C-reactive protein; NLR, neutrophil to lymphocyte ratio; PLR, platelet to lymphocyte ratio; SII, systemic immune-inflammation index; SIRI, systemic inflammatory response index; AISI, aggregate index of systemic inflammation; MII-1, multi-inflammatory index-Ⅰ; MII-2, multi-inflammatory index-II; MII-3, multi-inflammatory index-III.

All models were adjusted for age, sex, BMI, alcohol consumption, and log_2_-transformed triglyceride levels, with each log_2_-transformed inflammatory marker entered separately. Internal validation was performed using 2,000 bootstrap resamples.

**Figure 3:**
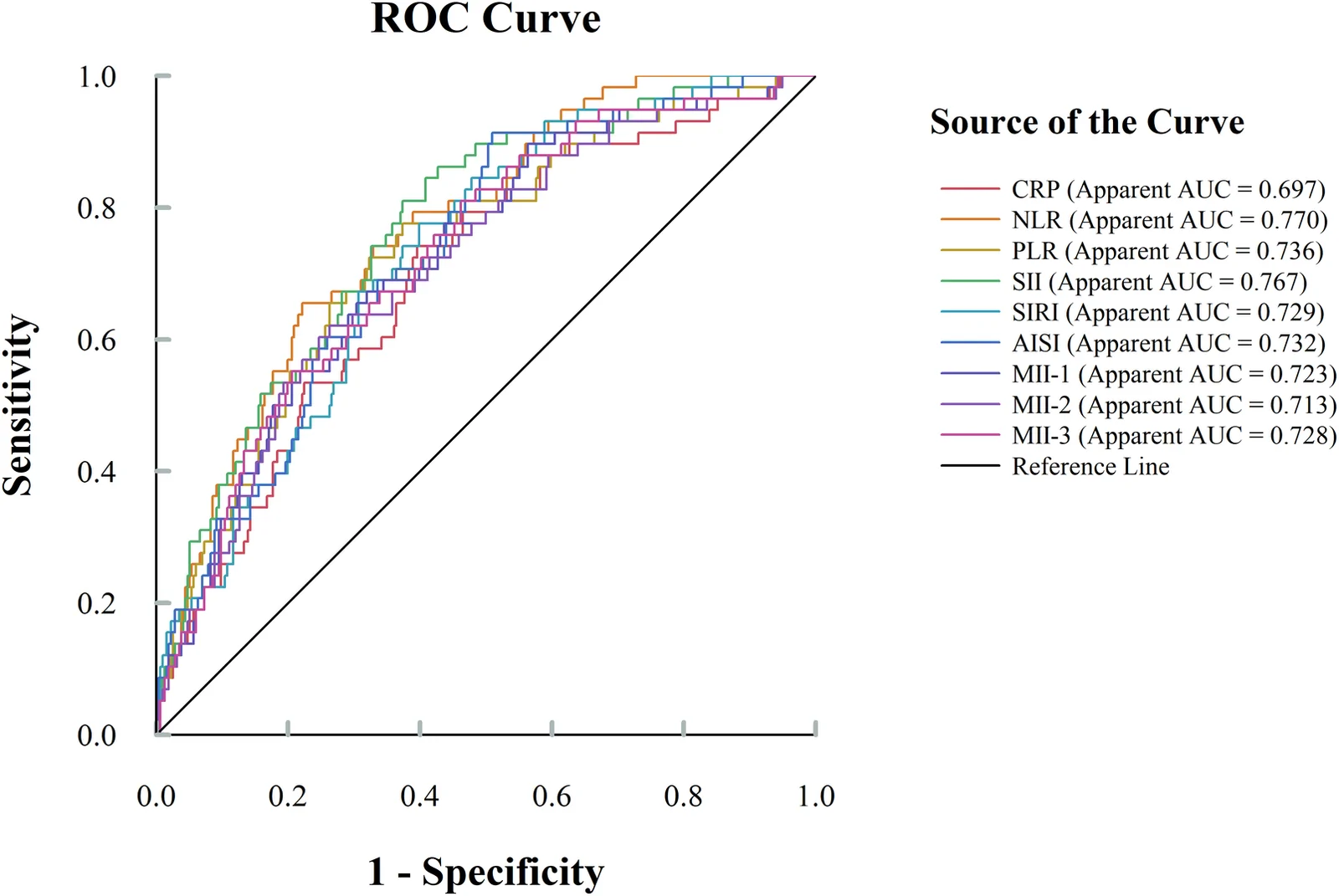
ROC curves for adjusted multivariable models for predicting SAP at admission. ROC, receiver operating characteristic; CRP, C-reactive protein; NLR, neutrophil to lymphocyte ratio; PLR, platelet to lymphocyte ratio; SII, systemic immune-inflammation index; SIRI, systemic inflammatory response index; AISI, aggregate index of systemic inflammation; MII-1, multi-inflammatory index-I; MII-2, multi-inflammatory index-II; MII-3, multi-inflammatory index-III.

At 48 h after admission, the adjusted models showed moderate-to-good discriminatory performance for assessing AP severity, with apparent AUCs ranging from 0.747 to 0.821. The NLR model showed the highest apparent AUC of 0.821, closely followed by the MII-1 model, which showed an AUC of 0.819. The MII-2 and MII-3 models also showed moderate-to-good discriminatory performance, with apparent AUCs of 0.807 and 0.804, respectively. After bootstrap internal validation, the optimism-corrected AUCs were 0.802 for NLR, 0.800 for MII-1, 0.786 for MII-2, and 0.783 for MII-3. The optimism-corrected calibration slopes ranged from 0.832 to 0.888, with values of 0.884, 0.872, and 0.874 for the MII-1, MII-2, and MII-3 models, respectively. These results indicate that the moderate-to-good discriminatory performance of the adjusted models incorporating MII markers for assessing AP severity at 48 h after admission was largely maintained after internal validation (Table 9 and Figure 4).

**Table 9:** Bootstrap internal validation of the adjusted models at 48 h after admission.

Variables	Apparent AUC	Estimated optimism	Optimism-corrected AUC	Optimism-corrected calibration slope
CRP	0.779	0.023	0.756	0.856
NLR	0.821	0.019	0.802	0.888
PLR	0.747	0.031	0.717	0.832
SII	0.789	0.024	0.765	0.867
SIRI	0.774	0.024	0.751	0.863
AISI	0.756	0.026	0.730	0.856
MII-1	0.819	0.019	0.800	0.884
MII-2	0.807	0.020	0.786	0.872
MII-3	0.804	0.021	0.783	0.874

AUC, area under the curve; CRP, C-reactive protein; NLR, neutrophil to lymphocyte ratio; PLR, platelet to lymphocyte ratio; SII, systemic immune-inflammation index; SIRI, systemic inflammatory response index; AISI, aggregate index of systemic inflammation; MII-1, multi-inflammatory index-Ⅰ; MII-2, multi-inflammatory index-II; MII-3, multi-inflammatory index-III.

All models were adjusted for age, sex, BMI, alcohol consumption, and log_2_-transformed triglyceride levels, with each log_2_-transformed inflammatory marker entered separately. Internal validation was performed using 2,000 bootstrap resamples.

**Figure 4:**
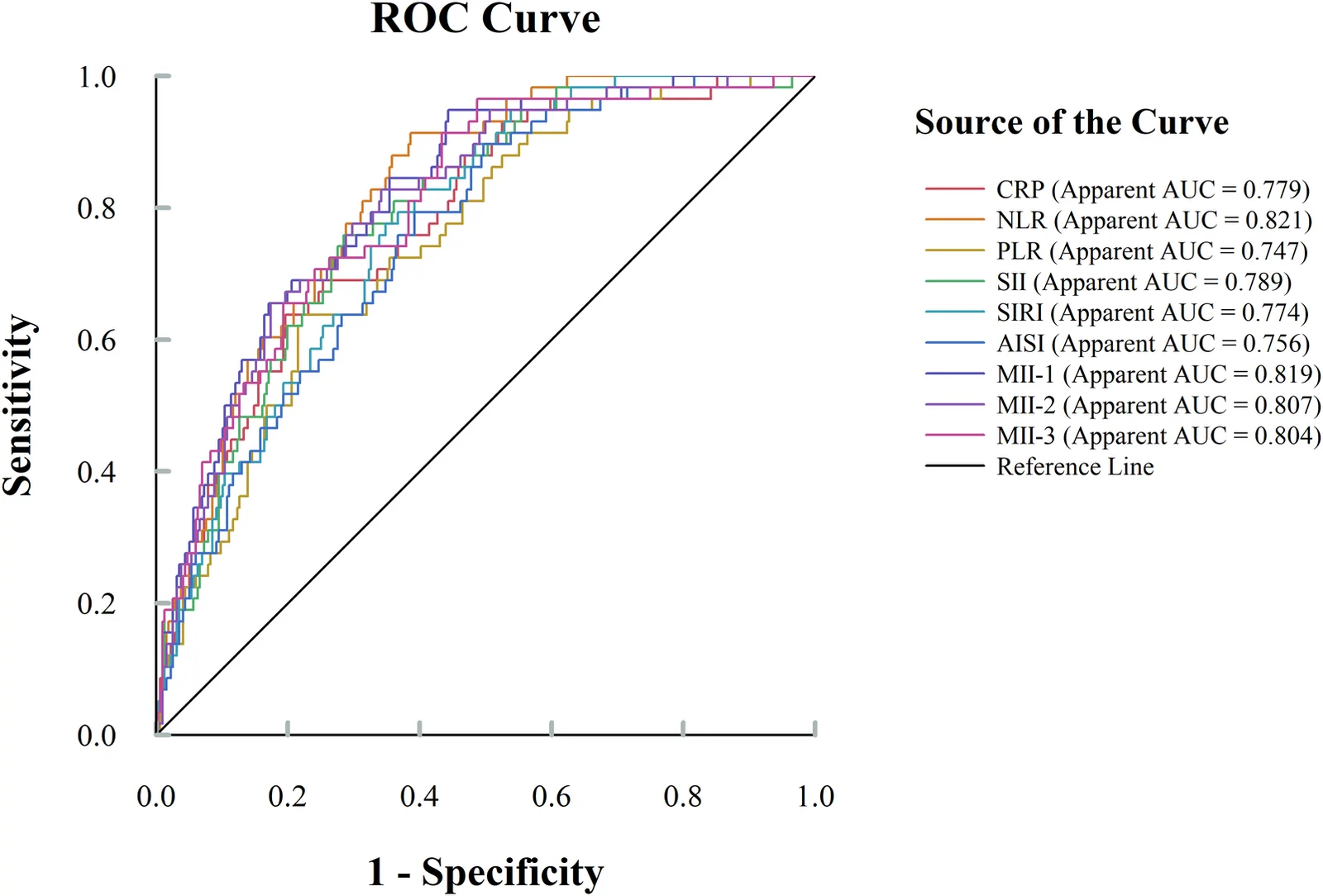
ROC curves for adjusted multivariable models for assessing AP severity at 48 h after admission. ROC, receiver operating characteristic; CRP, C-reactive protein; NLR, neutrophil to lymphocyte ratio; PLR, platelet to lymphocyte ratio; SII, systemic immune-inflammation index; SIRI, systemic inflammatory response index; AISI, aggregate index of systemic inflammation; MII-1, multi-inflammatory index-I; MII-2, multi-inflammatory index-II; MII-3, multi-inflammatory index-III.

## 4 Discussion

This study evaluated the clinical value of systemic inflammatory markers in predicting SAP at admission and assessing AP severity at 48 h after admission. AP, an inflammatory disease of the pancreatic tissue, is clinically characterized by sudden onset, unpredictable occurrence, and rapid progression. When the disease progresses to necrosis, infection of the pancreas or surrounding tissues, or multiple organ failure, the mortality rate rises to 20%–40% (4, 25). Therefore, assessment of AP severity is of great significance in determining the appropriate treatment plan. Given that the inflammatory markers SII, SIRI, AISI, and MII are readily accessible, easy to calculate, and significantly correlated with disease severity, they are likely to gain recognition as inflammatory markers for identifying SAP in future clinical practice.

The present study focused primarily on SII, SIRI, AISI, MII-1, MII-2, and MII-3, with CRP, NLR, and PLR included as reference inflammatory markers. All six primary indices were significantly higher in patients with SAP at admission and 48 h after admission. At admission, the AUCs of the six indices ranged from 0.620 to 0.694, with SII showing the highest value. At 48 h after admission, the AUCs ranged from 0.698 to 0.790, and MII-1, MII-3, and MII-2 yielded the three highest values. After adjustment for age, sex, BMI, alcohol consumption, and triglyceride levels, all six indices remained significantly associated with SAP. The discriminatory performance of the corresponding adjusted models was also largely preserved after bootstrap internal validation. Overall, these results indicate that the six inflammatory indices are associated with AP severity, with MII indices showing more moderate-to-good discriminatory performance at 48 h after admission.

The SII was first proposed by Hu et al. in 2014 and was initially used to predict the prognosis of patients after curative resection for hepatocellular carcinoma (12). Subsequently, Liu et al. first applied the SII to determine AP severity and found it more valuable than the neutrophil-to-lymphocyte ratio and the platelet-to-lymphocyte ratio (26). The SII is a marker based on neutrophil, lymphocyte, and platelet counts. Neutrophils play a role throughout the pathogenesis of AP, from local tissue damage to systemic inflammatory response. Under the influence of chemokines, neutrophils are rapidly recruited to the site of pancreatic injury to clear necrotic debris and control infection. However, excessive neutrophil activation becomes a key factor in pancreatic injury. Activated neutrophils release large amounts of proteolytic enzymes and reactive oxygen species, which directly attack and destroy acinar cells and damage vascular endothelial cells, further promoting the development of systemic inflammatory response syndrome. Neutrophil infiltration is not only the core mechanism of pancreatic injury itself but also the primary pathological basis for AP-associated acute lung injury, acute respiratory distress syndrome, and multi-organ dysfunction involving the liver, kidneys, and other organs (27). In contrast to the elevated white blood cell counts, lymphocyte counts are significantly reduced in SAP. Lymphocytes are an important component of immune regulatory cells and play several critical roles in AP onset and progression (28). Initially elevated as a result of systemic stress, they subsequently participate in mediating the inflammatory response. Their persistently low levels in peripheral blood can serve as an independent predictor of inflammatory progression, bacteremia, or sepsis, making them practical indicators for assessing the severity and prognosis of AP (29). In the pathophysiological process of AP, platelets serve as a bridge between inflammatory response and coagulation activation, and they are key to linking the inflammatory response with microcirculatory dysfunction. In patients with SAP, early thrombocytopenia may stem from microthrombus formation and subsequent platelet consumption. This is followed by reactive thrombocytosis characterized by an increase in high-volume platelets (L-PLT), which possess the highest procoagulant potential. More importantly, platelets undergo significant activation in AP. Cell surface adhesion molecules, such as the glycoproteins (Gp) Ⅱb/Ⅲa and P-selectin on the platelet surface, promote interactions between platelets and leukocytes and between platelets and endothelial cells. This not only facilitates microthrombus formation but also amplifies inflammation, creating a vicious cycle of coagulation and inflammation that leads to exacerbated acinar necrosis and multi-organ complications (30). This microthrombus-induced platelet depletion is offset by the body's compensatory platelet proliferation, making it impossible to determine the value of platelet count changes for identifying SAP. This finding is consistent with the absence of significant between-group differences in this study at admission and 48 h after admission. Liu et al. found that an SII >2,207.53 provides excellent guidance for predicting non-MAP, with an AUC of 0.920, a sensitivity of 92.9%, and a specificity of 87.7% (26). Zhang et al. reported significantly higher mortality rates in the moderate and high SII groups than in the low SII group, suggesting that higher SII values are correlated with more severe AP (25). In another recent study, SII was also used as an AP severity predictor, with an AUC of 0.615, a cutoff value of 1,860.35, a sensitivity of 0.80, and a specificity of 0.51 (31). In the present study, SII showed the highest AUC at admission (AUC = 0.694, 95% CI: 0.620–0.761), indicating moderate predictive value for SAP. At 48 h after admission, its AUC increased to 0.734, underscoring its increasing utility in assessing AP severity as the disease evolves. After adjustment for age, sex, BMI, alcohol consumption, and triglyceride levels, SII remained significantly associated with SAP.

The SIRI was first developed by Qi et al. in 2016 and was initially used to predict independent prognostic factors for survival in patients with pancreatic cancer who were receiving chemotherapy (13). Subsequently, Silva-Vaz et al. were the first to demonstrate that SIRI could serve as a novel prognostic indicator for AP severity, showing significant value in predicting the severity of acute biliary pancreatitis and 48 h mortality (32). SIRI is a marker based on neutrophil, lymphocyte, and monocyte counts. As core effector cells of the innate immune system, monocytes play a pivotal role in bridging the gap between innate and adaptive immunity. They not only participate directly in immune response as phagocytes and antigen-presenting cells but also coordinate the body's overall immune response to infection and inflammation (33). Wu et al. found an SIRI score of >9.5 (AUC = 0.67) to be predictive of SAP (34). In the present study, SIRI showed an AUC of 0.657 at admission, which increased to 0.700 at 48 h after admission. Notably, at 48 h after admission, SIRI showed the highest sensitivity of 0.931 among the evaluated inflammatory markers; however, its specificity was relatively low at 0.421. After adjustment for age, sex, BMI, alcohol consumption, and triglyceride levels, SIRI remained significantly associated with SAP at both time points. These findings underscore the utility of SIRI for identifying SAP patients at risk, particularly at 48 h after admission; however, its relatively low specificity should be considered.

The AISI was first established by Paliogiannis et al. in 2018, who identified that it holds significant value in predicting postoperative LOS in patients undergoing elective thoracotomy (35). AISI is based on a more comprehensive complete blood count, including neutrophil, lymphocyte, platelet, and monocyte counts. Subsequently, Zengin et al. first specifically applied AISI to the study of AP, showing that it is closely associated with AP severity and clinical outcomes (e.g., LOS and systemic and local complications) with high predictive accuracy (36). In the present study, AISI showed an AUC of 0.653 at admission and 0.698 at 48 h after admission. After adjustment for age, sex, BMI, alcohol consumption, and triglyceride levels, AISI remained significantly associated with SAP at both time points. However, its discriminatory ability as an individual marker was relatively limited, suggesting that AISI may be more appropriate as a supplementary indicator rather than a stand-alone marker of AP severity.

The MII-1, MII-2, and MII-3 were first established by Gardini et al. in 2020. Initial studies in patients with metastatic colorectal cancer indicated that all MII indices are prognostic biomarkers with clinical utility (14). Subsequently, Çinaroglu et al. noted that the MII-1, MII-2, and MII-3 can effectively predict the risk of walled-off pancreatic necrosis (WOPN) and mortality in patients with AP presenting to the emergency department (37). All of the three markers (MII-1, MII-2, and MII-3) have CRP as a common component. Due to its ease of detection, cost-effectiveness, and low technical barriers, CRP has become a commonly used biomarker for assessing conditions like inflammation, infection, venous thromboembolism, and cancer. As a circular protein comprising five identical subunits, its plasma concentration rises rapidly under pathological conditions like infection or tissue injury. CRP plays a crucial role in the body's defense mechanisms by activating the complement system, enhancing the phagocytic function of phagocytes to clear pathogens, and regulating cellular necrosis, apoptosis, and immune function. In the assessment of AP, CRP levels are widely used to determine disease severity: significantly elevated CRP typically indicates a more intense systemic inflammatory response and is associated with a higher risk of complications and adverse clinical outcomes (38). The 2025 IAP/APA/EPC/IPC/JPS guidelines indicate that a CRP level of >150 mg/L at admission has a sensitivity of 53% and specificity of 82% for predicting MSAP and SAP (3). Cardoso et al. showed that a CRP level of >190 mg/L at 48 h after admission was associated with a 20-fold higher probability of SAP compared to a level of >150 mg/L at the same time point (39). However, this marker also has certain limitations; for example, its blood concentration typically does not peak until 24–48 h after onset, and thus, a single early measurement has limited value (38). In the present study, CRP showed a high sensitivity of 0.966 at admission; however, its specificity and Youden index were only 0.190 and 0.155, respectively. Its AUC was also relatively low at 0.589, indicating that despite its high sensitivity, it had low specificity and limited overall discriminatory ability at admission. At 48 h after admission, the AUC of CRP increased to 0.758, with a sensitivity of 0.672 and specificity of 0.750. The improvement in the discriminatory performance of CRP from admission to 48 h after admission is consistent with its delayed increase during the inflammatory response. As CRP is incorporated into MII-1, MII-2, and MII-3, this shift may also partly explain the superior performance of the MII indices at 48 h after admission. At admission, MII-1 (AUC = 0.641, 95% CI: 0.572–0.708), MII-2 (AUC = 0.620, 95% CI: 0.547–0.694), and MII-3 (AUC = 0.648, 95% CI: 0.578–0.716) showed relatively limited predictive performance for SAP. At 48 h after admission, all three MII indices showed improved discriminatory performance for assessing AP severity, with MII-1 (AUC = 0.790, 95% CI: 0.728–0.845), MII-2 (AUC = 0.778, 95% CI: 0.718–0.835), and MII-3 (AUC = 0.780, 95% CI: 0.717–0.840) showing the three highest AUCs among the evaluated inflammatory markers. Pairwise DeLong comparisons further showed that—at 48 h after admission—MII-1, MII-2, and MII-3 had significantly higher AUCs than NLR, PLR, and SII, respectively. These findings suggest that combining CRP with the corresponding inflammatory indices improves their discriminatory performance for assessing AP severity compared with the indices alone. However, the AUCs of the three MII indices did not differ significantly from that of CRP, indicating that the contribution of CRP should also be considered when evaluating their moderate-to-good performance at 48 h after admission. The associations between MII indices and SAP remained significant after adjustment for age, sex, BMI, drinking, and triglyceride levels. At 48 h after admission, the adjusted models incorporating MII-1, MII-2, and MII-3 showed moderate-to-good discriminatory performance for assessing AP severity, with apparent AUCs of 0.819, 0.807, and 0.804, respectively. After bootstrap internal validation, the optimism-corrected AUCs were 0.800, 0.786, and 0.783, with the corresponding optimism-corrected calibration slopes being 0.884, 0.872, and 0.874, respectively. The minimal AUC optimism and calibration slopes remaining close to 1 indicate that the discriminatory performance and calibration of these models were largely preserved after internal validation. These findings further support the potential utility of MII indices for assessing AP severity at 48 h after admission. Although previous studies have shown the association of the MII-1, MII-2, and MII-3 with the incidence of WOPN and AP-associated mortality, evidence regarding their value for identifying SAP remains limited. In the present study, although the three MII indices showed relatively limited predictive performance for SAP at admission, they showed moderate-to-good discriminatory performance for assessing AP severity at 48 h after admission. These findings expand upon previous evidence regarding the clinical application of MII indices in AP and further clarify their potential utility for severity assessment.

The relative performance of NLR and MII indices differed between the analysis of individual markers and that of adjusted models. At 48 h after admission, all three MII indices showed higher AUCs than NLR when evaluated individually, and MII-1 showed a significantly higher AUC than NLR in the pairwise DeLong comparison. However, after adjustment for age, sex, BMI, alcohol consumption, and triglyceride levels, the NLR model showed the highest AUC at both time points. At 48 h after admission, the difference between the NLR and MII-1 models was minimal, with apparent AUCs of 0.821 and 0.819 and optimism-corrected AUCs of 0.802 and 0.800, respectively. These findings suggest that MII indices, particularly MII-1, showed moderate-to-good discriminatory performance for assessing AP severity when evaluated individually at 48 h after admission, whereas NLR also performed well when combined with clinical factors.

## 5 Limitations

This study has some limitations. First, this was a single-center retrospective study. Due to limited resources, we could not collect relevant patient data from multiple hospitals, which may limit its applicability across different clinical settings. Second, the exclusion of patients with a hospital stay of < 48 h may have introduced selection bias because complete data at 48 h after admission were unavailable for these patients. Among the six patients excluded for this reason, five showed clinical improvement and one showed clinical deterioration. Although the number was small, their exclusion may have affected the distribution of disease severity and discriminatory performance of the inflammatory markers. Besides, the limited number of patients with SAP (*n* = 58) may still pose a risk of overfitting despite restricting model complexity and performing bootstrap internal validation. Therefore, large-scale, multicenter, prospective studies are required to validate our findings and further evaluate the associations of these inflammatory markers with other aspects of patients' clinical status. Furthermore, direct comparisons between the inflammatory markers and established clinical scoring systems were not performed in this study. Further studies are needed to compare their performance with established scoring systems and assess whether these markers can contribute to models for AP severity assessment.

## 6 Conclusions

This study investigated the clinical value of the inflammatory markers SII, SIRI, AISI, MII-1, MII-2, and MII-3 in predicting SAP at admission and assessing AP severity at 48 h after admission. At admission, SII showed the highest predictive performance among the primary inflammatory markers. In particular, the present study further evaluated the potential value of the novel MII-1, MII-2, and MII-3 indices in AP severity assessment. At 48 h after admission, MII indices showed moderate-to-good discriminatory performance, particularly MII-1, and their associations with SAP remained significant after adjustment for clinical factors. These findings should be considered exploratory and hypothesis-generating, and further studies are needed to validate the potential value of MII indices in AP severity assessment.

## Data Availability

The raw data supporting the conclusions of this article will be made available by the authors, without undue reservation.
