# Supplementary material for: A clinical study on the application of new inflammatory markers SII, SIRI, AISI, and MII in the identification of severe acute pancreatitis
Source: Front Med (Lausanne). 2026 Sep 23;13:1880982. doi: 10.3389/fmed.2026.1880982 (PMC13642693; doi:10.3389/fmed.2026.1880982)
Supplement: Supplementary file 1 [file Table1.docx]

Supplementary Material

# 1 Supplementary Data

# 2 Supplementary Figures and Tables

Supplementary Table S1 Spearman Correlation Matrix at Admission.

| Variables | CRP | NLR | PLR | SII | SIRI | AISI | MII-1 | MII-2 | MII-3 |
| --- | --- | --- | --- | --- | --- | --- | --- | --- | --- |
| CRP | 1 |  |  |  |  |  |  |  |  |
| NLR | 0.205*** | 1 |  |  |  |  |  |  |  |
| PLR | 0.099 | 0.810*** | 1 |  |  |  |  |  |  |
| SII | 0.174*** | 0.901*** | 0.863*** | 1 |  |  |  |  |  |
| SIRI | 0.319*** | 0.769*** | 0.522*** | 0.764*** | 1 |  |  |  |  |
| AISI | 0.268*** | 0.678*** | 0.569*** | 0.823*** | 0.928*** | 1 |  |  |  |
| MII-1 | 0.941*** | 0.507*** | 0.357*** | 0.452*** | 0.534*** | 0.460*** | 1 |  |  |
| MII-2 | 0.953*** | 0.426*** | 0.376*** | 0.412*** | 0.454*** | 0.420*** | 0.981*** | 1 |  |
| MII-3 | 0.931*** | 0.498*** | 0.390*** | 0.502*** | 0.553*** | 0.527*** | 0.988*** | 0.983*** | 1 |

Abbreviations: CRP, C-reactive protein; NLR, neutrophil to lymphocyte ratio; PLR, platelet to lymphocyte ratio; SII, systemic immune-inflammation index; SIRI, systemic inflammatory response index; AISI, aggregate index of systemic inflammation; MII-1, multi-inflammatory index-Ⅰ; MII-2, multi-inflammatory index-II; MII-3, multi-inflammatory index-III.

*** Correlation is significant at the 0.001 level (two-tailed).

Supplementary Table S2 Spearman Correlation Matrix at 48 h After Admission.

| Variables | CRP | NLR | PLR | SII | SIRI | AISI | MII-1 | MII-2 | MII-3 |
| --- | --- | --- | --- | --- | --- | --- | --- | --- | --- |
| CRP | 1 |  |  |  |  |  |  |  |  |
| NLR | 0.489*** | 1 |  |  |  |  |  |  |  |
| PLR | 0.123* | 0.614*** | 1 |  |  |  |  |  |  |
| SII | 0.514*** | 0.869*** | 0.663*** | 1 |  |  |  |  |  |
| SIRI | 0.496*** | 0.871*** | 0.444*** | 0.799*** | 1 |  |  |  |  |
| AISI | 0.515*** | 0.766*** | 0.486*** | 0.904*** | 0.907*** | 1 |  |  |  |
| MII-1 | 0.872*** | 0.827*** | 0.392*** | 0.776*** | 0.766*** | 0.726*** | 1 |  |  |
| MII-2 | 0.868*** | 0.681*** | 0.541*** | 0.729*** | 0.612*** | 0.653*** | 0.912*** | 1 |  |
| MII-3 | 0.866*** | 0.774*** | 0.440*** | 0.850*** | 0.741*** | 0.801*** | 0.963*** | 0.936*** | 1 |

Abbreviations: CRP, C-reactive protein; NLR, neutrophil to lymphocyte ratio; PLR, platelet to lymphocyte ratio; SII, systemic immune-inflammation index; SIRI, systemic inflammatory response index; AISI, aggregate index of systemic inflammation; MII-1, multi-inflammatory index-Ⅰ; MII-2, multi-inflammatory index-II; MII-3, multi-inflammatory index-III.

* Correlation is significant at the 0.05 level (two-tailed).

*** Correlation is significant at the 0.001 level (two-tailed).

Supplementary Table S3 Pairwise DeLong Comparisons of MII-1, MII-2, and MII-3 With Other Inflammatory Markers at Admission.

| Variables | AUC of MII | Comparator | AUC of comparator | ΔAUC | P value |
| --- | --- | --- | --- | --- | --- |
| MII-1 | 0.641 | CRP | 0.589 | 0.052 | <0.001 |
| MII-1 | 0.641 | NLR | 0.686 | −0.045 | 0.284 |
| MII-1 | 0.641 | PLR | 0.632 | 0.009 | 0.848 |
| MII-1 | 0.641 | SII | 0.694 | −0.053 | 0.251 |
| MII-1 | 0.641 | SIRI | 0.657 | −0.016 | 0.719 |
| MII-1 | 0.641 | AISI | 0.653 | −0.012 | 0.807 |
| MII-2 | 0.620 | CRP | 0.589 | 0.031 | 0.024 |
| MII-2 | 0.620 | NLR | 0.686 | −0.066 | 0.150 |
| MII-2 | 0.620 | PLR | 0.632 | −0.012 | 0.799 |
| MII-2 | 0.620 | SII | 0.694 | −0.073 | 0.120 |
| MII-2 | 0.620 | SIRI | 0.657 | −0.037 | 0.432 |
| MII-2 | 0.620 | AISI | 0.653 | −0.033 | 0.505 |
| MII-3 | 0.648 | CRP | 0.589 | 0.058 | <0.001 |
| MII-3 | 0.648 | NLR | 0.686 | −0.038 | 0.362 |
| MII-3 | 0.648 | PLR | 0.632 | 0.016 | 0.723 |
| MII-3 | 0.648 | SII | 0.694 | −0.046 | 0.289 |
| MII-3 | 0.648 | SIRI | 0.657 | −0.009 | 0.831 |
| MII-3 | 0.648 | AISI | 0.653 | −0.005 | 0.911 |

Abbreviations: AUC, area under the curve; CRP, C-reactive protein; NLR, neutrophil to lymphocyte ratio; PLR, platelet to lymphocyte ratio; SII, systemic immune-inflammation index; SIRI, systemic inflammatory response index; AISI, aggregate index of systemic inflammation; MII-1, multi-inflammatory index-Ⅰ; MII-2, multi-inflammatory index-II; MII-3, multi-inflammatory index-III.

ΔAUC was calculated as the AUC of the MII index minus that of the comparator. P values were obtained using paired DeLong tests for correlated ROC curves. A positive ΔAUC indicates better discrimination by the MII marker than by the comparator.

Supplementary Table S4 Pairwise DeLong Comparisons of MII-1, MII-2, and MII-3 With Other Inflammatory Markers at 48 h after Admission.

| Variables | AUC of MII | Comparator | AUC of comparator | ΔAUC | P value |
| --- | --- | --- | --- | --- | --- |
| MII-1 | 0.790 | CRP | 0.758 | 0.032 | 0.119 |
| MII-1 | 0.790 | NLR | 0.742 | 0.048 | 0.017 |
| MII-1 | 0.790 | PLR | 0.645 | 0.145 | <0.001 |
| MII-1 | 0.790 | SII | 0.734 | 0.057 | 0.003 |
| MII-1 | 0.790 | SIRI | 0.700 | 0.090 | 0.002 |
| MII-1 | 0.790 | AISI | 0.698 | 0.092 | <0.001 |
| MII-2 | 0.778 | CRP | 0.758 | 0.020 | 0.370 |
| MII-2 | 0.778 | NLR | 0.742 | 0.036 | 0.225 |
| MII-2 | 0.778 | PLR | 0.645 | 0.132 | <0.001 |
| MII-2 | 0.778 | SII | 0.734 | 0.044 | 0.073 |
| MII-2 | 0.778 | SIRI | 0.700 | 0.078 | 0.038 |
| MII-2 | 0.778 | AISI | 0.698 | 0.079 | 0.016 |
| MII-3 | 0.780 | CRP | 0.758 | 0.022 | 0.245 |
| MII-3 | 0.780 | NLR | 0.742 | 0.038 | 0.139 |
| MII-3 | 0.780 | PLR | 0.645 | 0.135 | 0.002 |
| MII-3 | 0.780 | SII | 0.734 | 0.047 | 0.002 |
| MII-3 | 0.780 | SIRI | 0.700 | 0.080 | 0.014 |
| MII-3 | 0.780 | AISI | 0.698 | 0.082 | <0.001 |

Abbreviations: AUC, area under the curve; CRP, C-reactive protein; NLR, neutrophil to lymphocyte ratio; PLR, platelet to lymphocyte ratio; SII, systemic immune-inflammation index; SIRI, systemic inflammatory response index; AISI, aggregate index of systemic inflammation; MII-1, multi-inflammatory index-Ⅰ; MII-2, multi-inflammatory index-II; MII-3, multi-inflammatory index-III.

ΔAUC was calculated as the AUC of the MII index minus that of the comparator. P values were obtained using paired DeLong tests for correlated ROC curves. A positive ΔAUC indicates better discrimination by the MII marker than by the comparator.

Supplementary Table S5 Regression Coefficients of the Multivariable Logistic Regression Models at Admission.

| Variables | Age β | Sex β | BMI β | alcohol consumption β | Log_2_(TG) β | Inflammatory marker β | Intercept |
| --- | --- | --- | --- | --- | --- | --- | --- |
| CRP | 0.020 | −0.572 | 0.072 | 0.806 | 0.173 | 0.080 | −5.338 |
| NLR | 0.004 | −0.500 | 0.079 | 0.919 | 0.176 | 0.754 | −7.120 |
| PLR | 0.013 | −0.453 | 0.080 | 0.926 | 0.190 | 0.617 | −9.892 |
| SII | 0.013 | −0.432 | 0.079 | 0.981 | 0.160 | 0.657 | −12.389 |
| SIRI | 0.017 | −0.637 | 0.074 | 0.830 | 0.166 | 0.445 | −5.983 |
| AISI | 0.021 | −0.574 | 0.074 | 0.872 | 0.158 | 0.381 | −9.028 |
| MII-1 | 0.017 | −0.535 | 0.069 | 0.745 | 0.167 | 0.146 | −5.910 |
| MII-2 | 0.018 | −0.529 | 0.070 | 0.774 | 0.171 | 0.124 | −6.419 |
| MII-3 | 0.018 | −0.507 | 0.069 | 0.753 | 0.162 | 0.153 | −7.241 |

Abbreviations: CRP, C-reactive protein; NLR, neutrophil to lymphocyte ratio; PLR, platelet to lymphocyte ratio; SII, systemic immune-inflammation index; SIRI, systemic inflammatory response index; AISI, aggregate index of systemic inflammation; MII-1, multi-inflammatory index-Ⅰ; MII-2, multi-inflammatory index-II; MII-3, multi-inflammatory index-III.

Each row represents a separate multivariable logistic regression model including age, sex, BMI, alcohol consumption, log_2_-transformed triglyceride levels, and the indicated log_2_-transformed inflammatory marker. Sex was coded as male = 1 and female = 0; alcohol consumption was coded as yes = 1 and no = 0.

Supplementary Table S6 Regression Coefficients of the Multivariable Logistic Regression Models at 48 h After Admission.

| Variables | Age β | Sex β | BMI β | Alcohol consumption β | Log_2_(TG) β | Inflammatory  marker β | Intercept |
| --- | --- | --- | --- | --- | --- | --- | --- |
| CRP | 0.013 | −0.834 | 0.056 | 0.751 | 0.004 | 0.933 | −10.348 |
| NLR | −0.002 | −0.985 | 0.092 | 1.243 | 0.197 | 1.184 | −7.992 |
| PLR | 0.014 | −0.562 | 0.086 | 0.966 | 0.201 | 0.847 | −11.563 |
| SII | 0.009 | −0.804 | 0.071 | 1.126 | 0.167 | 0.756 | −12.334 |
| SIRI | 0.013 | −1.016 | 0.080 | 1.108 | 0.225 | 0.679 | −6.323 |
| AISI | 0.017 | −0.880 | 0.069 | 1.056 | 0.199 | 0.498 | −9.595 |
| MII-1 | 0.002 | −1.017 | 0.071 | 1.008 | 0.063 | 0.726 | −11.066 |
| MII-2 | 0.009 | −0.817 | 0.065 | 0.895 | 0.052 | 0.790 | −15.494 |
| MII-3 | 0.008 | −0.901 | 0.061 | 1.011 | 0.065 | 0.558 | −13.606 |

Abbreviations: CRP, C-reactive protein; NLR, neutrophil to lymphocyte ratio; PLR, platelet to lymphocyte ratio; SII, systemic immune-inflammation index; SIRI, systemic inflammatory response index; AISI, aggregate index of systemic inflammation; MII-1, multi-inflammatory index-Ⅰ; MII-2, multi-inflammatory index-II; MII-3, multi-inflammatory index-III.

Each row represents a separate multivariable logistic regression model including age, sex, BMI, alcohol consumption, log_2_-transformed triglyceride levels, and the indicated log_2_-transformed inflammatory marker. Sex was coded as male = 1 and female = 0; alcohol consumption was coded as yes = 1 and no = 0.

Supplementary Table S7 Maximum Variance Inflation Factors of the Multivariable Logistic Regression Models.

| Inflammatory marker | Maximum VIF at admission | Maximum VIF at 48 h after admission |
| --- | --- | --- |
| CRP | 1.430 | 1.534 |
| NLR | 1.540 | 1.555 |
| PLR | 1.475 | 1.449 |
| SII | 1.453 | 1.455 |
| SIRI | 1.434 | 1.451 |
| AISI | 1.420 | 1.424 |
| MII-1 | 1.462 | 1.504 |
| MII-2 | 1.447 | 1.470 |
| MII-3 | 1.445 | 1.464 |

Abbreviations: VIF, variance inflation factors; CRP, C-reactive protein; NLR, neutrophil to lymphocyte ratio; PLR, platelet to lymphocyte ratio; SII, systemic immune-inflammation index; SIRI, systemic inflammatory response index; AISI, aggregate index of systemic inflammation; MII-1, multi-inflammatory index-Ⅰ; MII-2, multi-inflammatory index-II; MII-3, multi-inflammatory index-III.

At each time point, the maximum VIF represents the highest VIF among all variables included in the corresponding multivariable logistic regression model.
